# Small-Diameter PTFE Portosystemic Shunts: Portocaval vs Mesocaval

**DOI:** 10.1155/1998/67507

**Published:** 1998

**Authors:** Robert Shields

**Affiliations:** The Royal College of Surgeons of Edinburgh Nicolson Street Edinburgh EH8 9DW United Kingdom

## Abstract

Fifty-seven patients with failed sclerotherapy received a mesocaval interposition shunt with an externally supported, ringed polytetrafluoroethylene prosthesis of either 10 or 12 mm diameter. Thirty-one patients had Child-Pugh gradeA disease and 26 grade B; all had a liver volume of 1000– 2500 ml. Follow-up ranged from 16 months to 6 years 3 months. Three patients (5 per cent) died in the postoperative period. There were two postoperative recurrences of variceal haemorrhage and one recurrent bleed in the second year after surgery. The cumulative shunt patency rate was 95 per cent and the incidence of encephalopathy 9 per cent; the latter was successfully managed by protein restriction and/or lactulose therapy. The actuarial survival rate for the whole group at 6 years was 78 per cent, for those with Child-Pugh grade A 88 per cent and for grade B 67 per cent. Small-lumen mesocaval interposition shunting achieves portal decompression, preserves hepatopetal flow, has a low incidence of shunt thrombosis, prevents recurrent variceal bleeding and is not associated with significant postoperative encephalopathy.


Ellison syndrome in multiple endocrine neoplasia. *Surgery*, **114**, 1183 – 1188.
[3] Radebold, K., Borman, P. C., van Wyk, M. E. C., Krige, J. E. J., Mostert, C. and Terblanche, J. (1996). Clinicopathological characteristics and prognosis of liver metastases in patients with Zollinger – Ellison syndrome. *Gastroenterology*, **110**, A1100.
[4] Zollinger, R. M. (1985). Gastrinoma: factors influencing prognosis. *Surgery*, **97**, 49 – 54.
[5] Fraker, D. L., Norton, J. A., Alexander, H. R., Venzon, D. J. and Jensen, R. T. (1994). Surgery in Zollinger – Ellison syndrome alters the natural history of gastrinoma. *Ann Surg*, **220**, 320 – 330.
[6] UICC International Union Against Cancer. TNM classification of malignant tumours (1987). Springer Verlag Berlin Heidelberg New York.

Professor P. C. Bornman
Head: Surgical Gastroenterology and
Gastrointestinal Clinic E23

K. Radebold, M.D.
Consultant Surgeon
Department of Surgery
University of Cape Town Medical School
Observatory 7925
Cape Town
South Africa

### Small-Diameter PTFE Portosystemic Shunts: Portocaval vs Mesocaval

### ABSTRACT

*Paquet, K.-J., Lazar, A., Koussouris, P., Hotzel, B., Gad, H. A., Kuhn, R. and Kalk, J.-F. (1997) Mesocaval interposition shunt with small-diameter polytetrafluoroethylene grafts in sclerotherapy failure, British Journal of Surgery; 82, 199 – 203.*

**Fifty-seven patients with failed sclerotherapy received a mesocaval interposition shunt with an externally supported, ringed polytetrafluoroethylene prosthesis of either 10 or 12 mm diameter. Thirty-one patients had Child-Pugh grade A disease and 26 grade B; all had a liver volume of 1000 – 2500 ml. Follow-up ranged from 16 months to 6 years 3 months. Three patients (5 per cent) died in the postoperative period. There were two postoperative recurrences of variceal haemorrhage and one recurrent bleed in the second year after surgery. The cumulative shunt patency rate was 95 per cent and the incidence of encephalopathy 9 per cent; the latter was successfully managed by protein restriction and/or lactulose therapy. The actuarial survival rate for the whole group at 6 years was 78 per cent, for those with Child-Pugh grade A 88 per cent and for grade B 67 per cent. Small-lumen mesocaval interposition shunting achieves portal decompression, preserves hepatopetal flow, has a low incidence of shunt thrombosis, prevents recurrent variceal bleeding and is not associated with significant postoperative encephalopathy.**

*Keywords:* Portocaval shunt, mesocaval shunt, Sarfeh shunt, narrow-diameter shunt

### PAPER DISCUSSION

The place of surgical operations in the treatment of portal hypertension has become much more circumscribed. For the most part, initial treatment, both emergency and elective, is nonsurgical and involves a direct attack on the varices, either sclerotherapy or banding. Balloon tamponade and vasoactive drugs represent adjuvant treatment only. Surgery is used when sclerotherapy or banding fails. Because of the post-operative incidence of hepatic failure and encephalopathy, end-to-side portacaval shunt was abandoned in favour of operations aimed to maintain prograde portal flow and hepatic perfusion, e.g. distal splenorenal shunt, small-diameter side-to-side portacaval shunts, etc. The value of maintaining hepatic perfusion remains largely theoretical. The operation of mesocaval interposition shunt had the advantage of technical simplicity. However, although the initial results seemed good, long-term outcome was not so optimistic in terms of rebleeding and encephalopathy. Graft thrombosis was common.

The authors of this paper report a 5-year study on 57 patients, using a polytetrafluoroethylene (PTFE), small-diameter graft between the superior mesenteric vein and the inferior cava. These operations were carried out when sclerotherapy had failed, failure being defined as two recurrences of bleeding from the oesophageal varices or one recurrence from gastric varices. The reported results seem good, with only 3 deaths (5%) in the post-operative period, and only 2 recurrences of haemorrhage in the immediate post-operative period and one recurrent later bleed in the second year after operation. The incidence of encephalopathy was reported as 9 per cent, and was managed successfully by protein restriction and/or lactulose treatement. The cumulative patency rate of the shunt was 95 per cent. There was a 75 per cent actuarial survival rate at 6 years.

The patients were extensively investigated before, and after, operation. The operative techniques were standard apart from the use of a PTFE graft. The patients were put on to heparin initially intravenously and later subcutaneously, to maintain a clotting level of twice normal. Significant reduction in portal pressure was achieved with shunt diameters of 10 and 12 millimetres. Prograde hepatic flow was preserved for at least 3 years. Chronic encephalopathy did not feature after shunting, but detailed description of the results of pre- and post-operative tests for encephalopathy were not provided.

The main criticism of the paper is that the study was restricted to patients who had good liver function, mainly Child-Pugh grade A and B. In the experience of most clinicians in this field, the majority of patients belong to the Child-Pugh grade C. How did the authors treat those in Child-Pugh grade C? Moreover, the authors do not describe how they assessed the severity of haemorrhage preceding these operations. Were these severe haemorrhages or mild, but repeated, bleeds? There is good evidence that the incidence of hepatic failure and encephalophy after any type of shunt is remarkably low in patients who have good liver function and who have not bled severely.

There is no doubt that the interposition mesocaval shunt is the easiest of all shunts to perform. The shunt can be easily taken down if, and when, liver transplant has to be undertaken. The authors have an extensive experience in this field, and have contributed significantly to the surgical literature. However, since the early days of end-to-side portacaval shunt, the tale has always been the same. The first reports of a new treatment have been most encouraging. The initial studies have been undertaken in a small number of highly-selected patients by an enthusiastic dedictated group. Only when the new treatment has been undertaken in larger numbers, in a less selected group of patients, and reported by a variety of groups, has pessimism set in.

The use of PTFE graft for mesocaval interposition, therefore, is a useful technique for the surgeon to keep at the back of his mind, for it is a simple technique when the superior mesenteric vein and the inferior vena cava are both patent. The results of this paper apply only to a small proportion of patients with bleeding oesophageal varices, and it has not been proved that the results are better than other forms of shunt. The authors are invited to undertake a randomised control trial comparing this technique with other established procedures.

Professor Sir Robert Shields
Oce of the President
The Royal College of Surgeons
of Edinburgh
Nicolson Street
Edinburgh EH8 9DW
United Kingdom

